# Isotopic evidence for soil water sources and reciprocal movement in a semi-arid degraded wetland: Implications for wetland restoration

**DOI:** 10.1016/j.fmre.2022.11.001

**Published:** 2022-11-17

**Authors:** Yuanchun Zou, Sijian Zhang, Xiaofei Yu, Guobin Fu, Xianguo Lu

**Affiliations:** a Heilongjiang Xingkai Lake Wetland Ecosystem National Observation and Research Station & Key Laboratory of Wetland Ecology and Environment & Jilin Provincial Joint Key Laboratory of Changbai Mountain Wetland and Ecology, Northeast Institute of Geography and Agroecology, Chinese Academy of Sciences, Changchun 130102, China; b State Environmental Protection Key Laboratory for Wetland Conservation and Vegetation Restoration & Jilin Provincial Key Laboratory of Ecological Restoration and Ecosystem Management & Key Laboratory of Vegetation Ecology of Ministry of Education, School of Environment, Northeast Normal University, Changchun 130117, China; c CSIRO Land and Water, Private Bag 5, Wembley, WA 6913, Australia

**Keywords:** Water stable isotopes, Unsaturated soil water sourcing, HYDRUS-1D, Degraded wetland restoration, Songnen Plain

## Abstract

Understanding water dynamics is a prerequisite for the restoration of degraded ecosystems in arid and semiarid regions. In this study, we carried out δD and δ^18^O analyses of precipitation, unsaturated soil water, overland flow, surface runoff, and groundwater samples from a seasonally flooded wetland in the Momoge National Nature Reserve of the Songnen Plain, Northeast China, to identify the water sources and understand the mechanisms of unsaturated soil water movement. Unsaturated soil water content (W/W%) at every 20 cm along with a soil profile (0–100 cm) was collected during the growing season, and the HYDRUS-1D model was used to simulate temporal-spatial variations. The results showed that the local meteoric water line (δD = 5.90δ^18^O-7.34, R^2^ = 0.95) had a smaller slope and intercept than the global meteoric water line because of strong evaporation at our study site under semi-arid climate. The groundwater was partly recharged by local precipitation *via* overland flow and unsaturated soil water infiltration. Unsaturated soil water was sourced from both precipitation and groundwater with variations at different depths. The upper soil layer at 0–15 cm was mainly sourced from limited precipitation, while the groundwater could move up to a 25 cm layer during the dry period. The unsaturated soil water content increased with soil depth in the top 40 cm, decreased at depths of 40 to 80 cm, and increased again at depths of 80 to 100 cm. The HYDRUS-1D model could simulate the unsaturated soil water dynamics well in the upper (0–40 cm) and lower (80–100 cm) sections, but poorly for depths of 40–80 cm due to the upward and downward flow. The bidirectional unsaturated soil water movement highlights the importance of capillary groundwater for wetland plants with similar climatic or hydrogeological conditions.

## Graphical abstract

**Figure:**
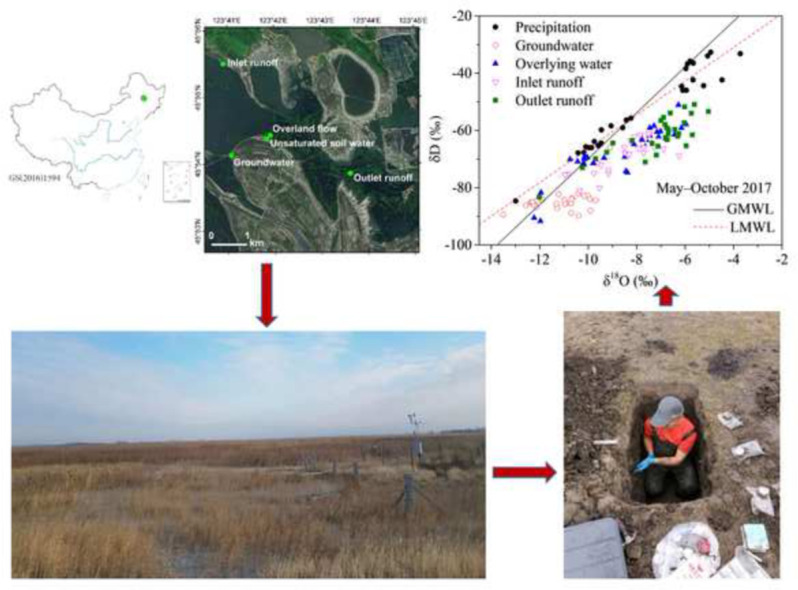

## 1 Introduction

As one of the key constituents of soil, water plays an important role in ecological processes and is crucial for life on Earth [1]. Unsaturated soil water movement is considered a major driving force of terrestrial ecosystems [2]. Identifying water sources and understanding the mechanisms of unsaturated soil water movement are prerequisites for the reconstruction and restoration of degraded ecosystems, especially in arid and semi-arid areas.

Stable oxygen and hydrogen isotopes, which naturally exist in water without extra artificial substances, can provide insight into subsurface hydrologic processes, such as the water source, flow pattern, velocity, and mean residence time [3,4,[5], [6], [7], [8], [9], [10], [11]]. Therefore, considerable water isotopic studies have been conducted in various ecosystems under different climatic conditions, including arid, semiarid, and humid conditions. Two major unsaturated soil water movement mechanisms, piston flow and preferential flow, have been proposed and confirmed in the unsaturated zone [6,12]. Given the groundwater recharge functioning of wetlands, it could be inferred that a better understanding of water movement from precipitation to groundwater *via* wetland soil profiles could be obtained by seasonal and vertical variations of stable isotope contents in precipitation, unsaturated soil water, and groundwater [13]. Such data could provide valuable information on the movement from precipitation to groundwater *via* the soil profile [7,14,10,15,16]. However, the differences in the isotopic composition of wetland surface water could reveal horizontal flow patterns, either in constructed wetlands [17] or in river-wetland systems [18].

Although the stable isotopic method has been successfully used to identify water sources for wetlands [19], [20], [21], the lack of coupled observation and prediction of unsaturated soil water has impeded the water-saving restoration of degraded wetlands caused by water shortages. Comprehensive wetland models (e.g. WETLANDS, MIKE SHE, CH3D, WASH123, and INHM) usually require long-term and macroscopic data, making it difficult to determine the unsaturated soil water source, movement, and distribution without sufficient historical data in time [22]. HYDRUS models, developed by the United States Saline Soil Laboratory and US National Salt Reform center, can be used to predict unsaturated soil water distribution using less multisource data [23].

Wetlands in semi-arid areas are expected to experience special unsaturated soil water movement and distribution. In this study, the oxygen and hydrogen isotopes of precipitation, wetland surface water, unsaturated soil water, and groundwater were observed, and the vertical distribution of unsaturated soil water was modelled in the Momoge National Nature Reserve (MNNR), a Ramsar site with a semi-arid climate that is subject to degradation caused by water shortage [24]. The objectives of this study were (1) to identify the water sources at different depths of wetland soil, (2) to understand the vertical distribution and movement of unsaturated soil water, and (3) to explore the critical timing of water replenishment.

## 2 Materials and methods

### 2.1 Study area

Located in the western Songnen Plain of Northeastern China along the Nenjiang River, MNNR (45°42′–46°18′ N, 123°27′–124°04′ E) includes various types of wetlands (e.g., marshes, shallow lakes, and floodplains), dominated by *Scirpus* spp., *Carex* spp., *Typha* spp., *and Phragmites australis*. It is one of the most important stopover sites for *Grus leucogeranus* globally, and the tuber of *S. planiculmis* is one of the most important food sources for *G. leucogeranus*. MNNR has a semi-arid monsoon climate, with an annual average temperature of 4.4 °C, precipitation of 380 mm, and evaporation of 1472 mm [25]. For agricultural water diversion and drainage, hydrological regimes have shifted from climate-driven to anthropogenic-driven. Consequently, some peripheral natural wetlands have degraded, and the primary wetland plants growing in shallow water have evolved from *S. planiculmis* to *T. angustifolia* and *P. australis*. To block surface water and create suitable foraging habitats for these migratory cranes, a loop causeway (ca. 4 *m* × 1 *m* × 36 km) was constructed around one of the key wetlands, White Crane Lake (formerly named Etoupao), in 2008. White Crane Lake is mainly supplied by precipitation in summer and discharge from upstream paddy fields in autumn *via* the unique water inlet in the northwest corner and flowing out through the outlet in the south causeway [26,27].

### 2.2 Water sampling and isotope analyses

**Fig. 1:**
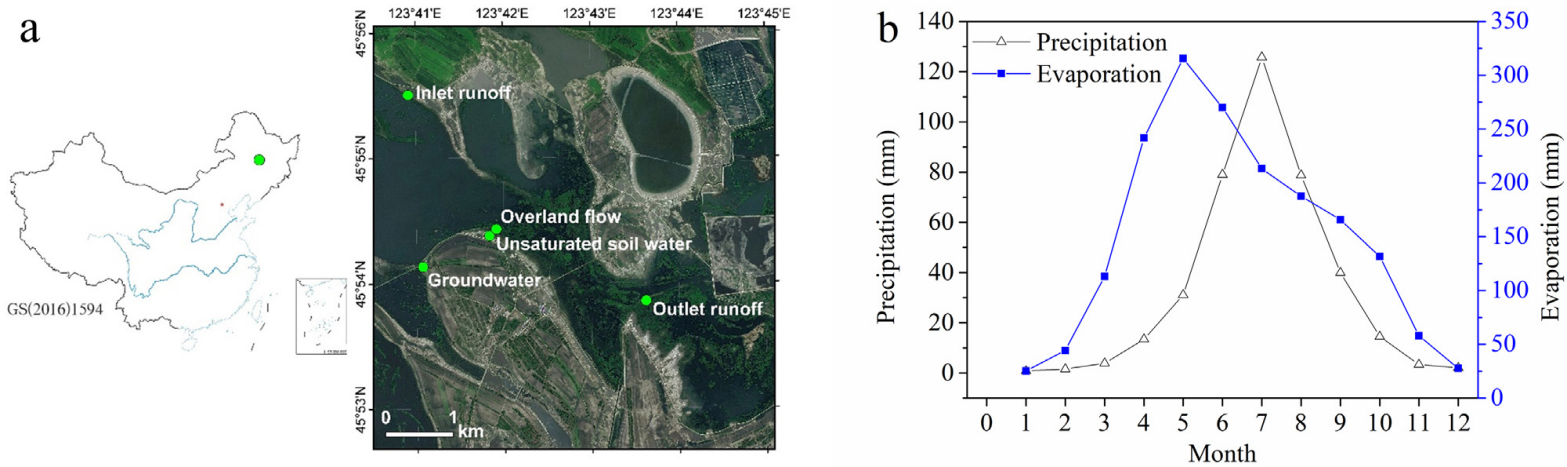
**Location map (a) and reference monthly mean precipitation and evaporation from 1960 to 2020 (b) of the study sties**.

The sampling area (45°53′–45°56′ N, 123°40′–123°44′ E) is located near the White Crane Lake Observatory (Fig. 1a). As there were no local weather stations for long-term monitoring, we obtained the monthly mean precipitation and evaporation from 1960 to 2020 at the Baicheng National Basic Weather Station as a reference (Fig. 1b). The marsh soil is seasonally flooded, with fluctuating surface water depths ranging from 0 to 60 cm, and the dominant plants are *P. australis* and *T. angustifolia*.

A weather station (HOBO U30, Onset, USA) was set up nearby to monitor the real-time weather data (e.g. rainfall, evapotranspiration, and temperature) and unsaturated soil water contents of the 0–20 cm, 20–40 cm, 40–60 cm, 60–80 cm, and 80–100 cm layers. In addition, another nearby soil column was collected in advance to test the soil texture compositions and bulk densities, layer by layer.

Unsaturated soil water extractors with negative pressure pipes were installed one month before sampling at 5, 15, 25, 35, 45, 55, 65, 75, 85, and 100 cm. Wetland unsaturated soil water samples were collected from each layer with three replicates from 21 June after the soil thawed thoroughly to 25 September before being frozen again in 2017. It is noteworthy that not all unsaturated soil water could be collected on each date. The equipment and sampling procedures can be found in Zou et al. [28].

Meanwhile, the wetland overland flow near the site, surface water from the inlet and outlet runoff, and groundwater in the vicinity of the study site were collected. Precipitation samples were collected in a plastic bottle using a funnel at the study site. A film of paraffin oil was used to prevent evaporation from the plastic bottle before collection.

Each water sample was then collected using a 0.45 μm needle filter and placed into a 2-mL vial. The bubbles were removed to prevent water evaporation fractionation, and all samples were stored in ice packs, brought back to the laboratory, and stored in freezers before measurement. The δD and δ^18^O values of all water samples were analysed using a stable isotope ratio mass spectrometer (MAT253), with analytical precisions of ±0.5‰ and ±0.2‰, respectively. The δD and δ^18^O values were reported relative to Vienna Standard Mean Ocean Water (VSMOW).

### 2.3 HYDRUS-1D modelling

The unsaturated soil water characteristics of the experimental plots at different times were simulated using the HYDRUS-1D model, as follows [23]:

(1) Calibrating soil hydraulic parameters. Soil bulk density of each layer was measured using the core method. The soil texture composition was measured using a laser diffraction analyser (Malvern Mastersizer 2000, UK). These data were then input into the neural network model provided by the Rosetta module of HYDRUS-1D. The unsaturated soil water characteristic curve parameters for each layer were calibrated to achieve the minimum difference between the simulated and field-recorded values. (2) Modelling the external set of conditions. The simulation time was 100 d, and daily rainfall and transpiration were input during the simulation. The Penman-Monteith equation was used to input the monitored meteorological data obtained from the field for further calculations. Potential evapotranspiration data were substituted into the HYDRUS-1D model to obtain atmospheric boundary conditions. (3) Stratifying the soil profile. A one-dimensional soil profile of 100 cm, with every 20 cm layer, was set for layering. The middle point of each soil layer was set as the model observation point at depths of 10, 30, 50, 70, and 90 cm. (4) Setting the model boundary. The upper boundary was set as the atmospheric environment boundary. For deeper groundwater levels (or if there is a thick clay layer), the exchange of surface water and groundwater resources is relatively rare. Therefore, the lower boundary of the simulated experimental wetland was set as the known groundwater level. (5) Determining the time step interval and dividing the physical soil grid structure. The time unit was set to 1 d, with a total simulation time of 100 d. The initial, minimum, and maximum simulation time intervals were 0.1, 0.01, and 5 days, respectively. The ideal cycle length for the model simulation was 3–7 days, and the maximum number of simulation cycles was 10. In addition, the allowable deviation in the unsaturated soil water content was set as 0.001, and the allowable deviation in the pressure head value was set as 1. (6) Validating the results of the model simulation. When processing the model, the empirically recommended root-mean-square error (RMSE) and determination coefficient (R^2^) were used as nonlinear simulation variables to evaluate the accuracy of the model-simulated results. After entering the relevant data and setting the parameters related to the external conditions of the model, the simulated unsaturated soil water content and measured values were compared, and whether the RMSE and R^2^ values met the simulation accuracy requirements was analysed.

### 2.4 Statistical analyses

One-way analysis of variance (ANOVA) was performed using IBM SPSS Statistics for Windows Version 21.0 (IBM Corp., USA) to test the temporal change effect on the δD and δ^18^O from the precipitation, groundwater, overland flow, and inlet and outlet runoff. Two-way ANOVA was used to test the main effects of temporal change and soil layer and their interaction effects on the δD, δ^18^O, and deuterium excess (d-excess, i.e. δD-8δ^18^O) from unsaturated soil waters. Pearson's correlation analysis was used to calculate the correlation coefficients (r) of δD and δ^18^O in various water samples. The local meteoric water line (LMWL), local groundwater line (LGWL), local unsaturated soil water lines (LUSWL), and all graphics were drawn using Origin Pro 9.0 (OriginLab Corp., USA). The means and standard errors as well as the coefficients of variation (CV,%) of δD and δ^18^O in different waters were calculated using Origin Pro 9.0.

## 3 Results

### 3.1 Isotopic compositions in precipitations, groundwater, and surface waters

**Fig. 2:**
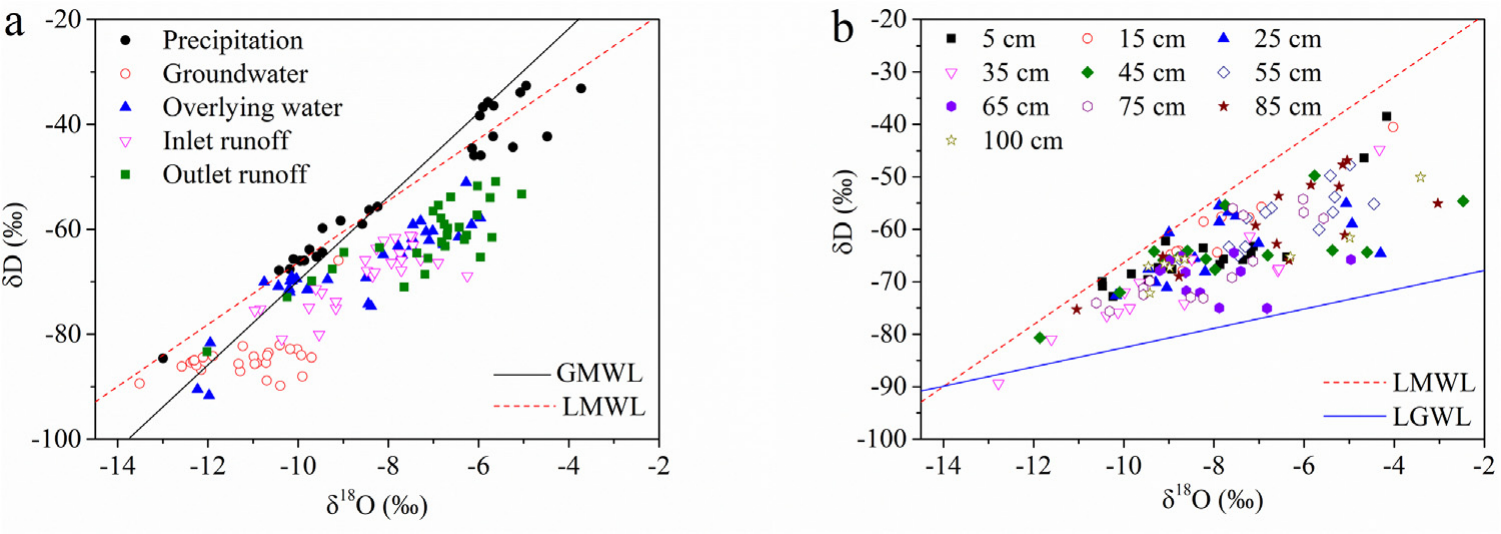
**δD and δ^18^O ranges of precipitation, groundwater, wetland overland flow, runoff (a), and soil water collected from different layers (b) from a typical degraded wetland in the Momoge National Nature Reserve (MNNR) during the growing season from June to September 2017.** The local meteoric water line (LMWL) was fitted by a linear regression equation, δD = 5.90δ^18^O-7.34 (R^2^ = 0.95). The local groundwater line (LGWL) was fitted using a linear regression equation as δD = 1.84δ^18^O-64.16 (R^2^ = 0.18). For reference, the global meteoric water line (GMWL) was defined as δD = 8δ^18^O+10.

**Fig. 3:**
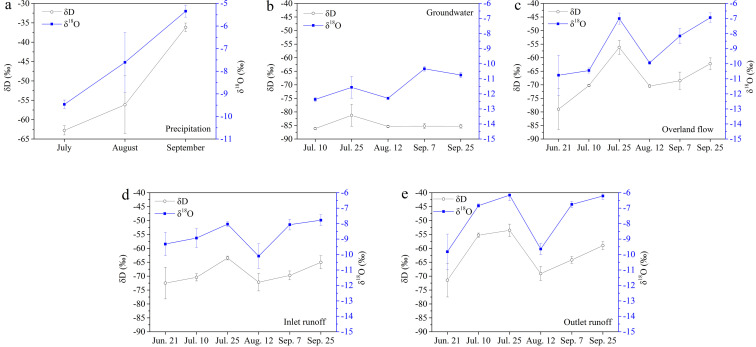
**Temporal changes of δD and δ^18^O in precipitation (a), groundwater (b), overland flow (c), and inlet (d) and outlet runoff (e)**.

According to the one-way ANOVA, δD had significant temporal variations in precipitation, wetland overland flow, and outlet runoff; however, the δ^18^O of all water samples showed significant variations (Table S1). The LMWL was fitted by a linear regression equation as δD = 5.90δ^18^O-7.34 (R^2^ = 0.95), with a smaller slope and intercept than that of the global meteoric water line (GMWL). The δD of precipitation varied from −85.4‰ to −32.7‰, and δ^18^O varied from −13.0‰ to −3.7‰. The isotopic compositions of groundwater were distributed in the lower left part, whereas those of the overlying wetland and surface waters showed heavier isotopic compositions without clear boundaries between each other (Fig. 2a). When the soil depth was considered, nearly all unsaturated soil water occurred within the LMWL and LGWL, except for one data point in the soil layer at a depth of 35 cm (Fig. 2b). The unsaturated soil water lines for each layer are listed in Table S2. Both δD and δ^18^O increased from July to September, whereas those of the groundwater, wetland overland flow, and inlet and outlet runoff were approximately sinusoidal curves (Fig. 3).

### 3.2 Spatiotemporal isotopic compositions of unsaturated soil waters

**Fig. 4:**
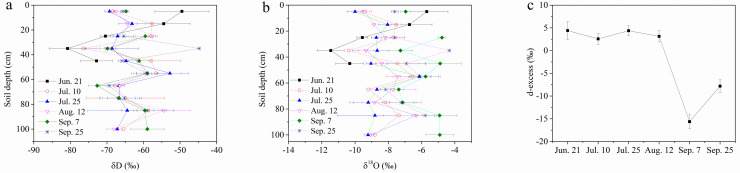
**Temporal changes of δD (a), δ^18^O (b), and deuterium excess (d-excess, c) in the soil water of each layer**. The d-excess was defined as d-excess = δD-8δ^18^O. Individual data were missed for the failure of soil water collection.

According to the two-way ANOVA, the main and interaction effects of temporal change and soil layer were significant for δD and δ^18^O, while the soil layer had a non-significant effect on d-excess (Table S3). The spatiotemporal curves of δD and δ^18^O varied and crossed each other (Fig. 4a, b). The d-excess remained stable in summer (June–August) and decreased in September (Fig. 4c). It is noteworthy that some data were missing for the failure of unsaturated soil water collection.

**Fig. 5:**
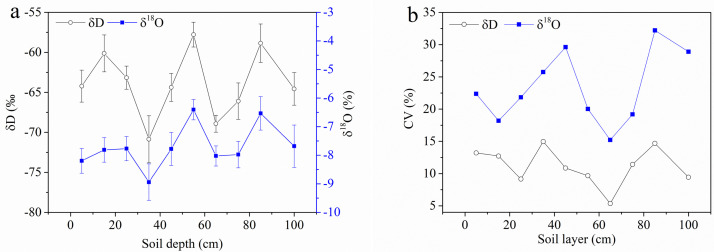
**Mean values of δD and δ^18^O (a), and the absolute coefficients of variation (CV, b) of the δD and δ^18^O in the soil water of each layer**.

For the temporal mean values of δD and δ^18^O, similar fluctuating trends could be observed; three peaks occurred in the 15-cm layer, 55-cm layer, and 85-cm layer respectively, while the two bottoms occurred in the 35-cm layer and 65-cm layer respectively (Fig. 5a). When all variations of different soil layers were considered, their absolute CV showed similar trends: the greatest values occurred in the 65-cm layer while the smallest ones in the 85-cm layer (Fig. 5b).

### 3.3 Determination of the main hydraulic parameters in the model

Soil texture composition and bulk density are shown in Table S4. Unsaturated soil parameters were identified using the model presented in Table S5. The unsaturated soil water content errors and actual measured values for each layer after multiple corrections of the model parameters are listed in Table S6. The RMSE of the unsaturated soil water in each layer varied from 0.00714 to 0.01964 cm^3^ cm^−3^, and the R^2^ varied from 0.68 to 0.86. The results showed that the measured and simulated values were in agreement in the 0–40 cm and 8–100 cm layers, while the consistency in the 40–80 cm layer was relatively weak.

### 3.4 Unsaturated soil water content modelling

**Fig. 6:**
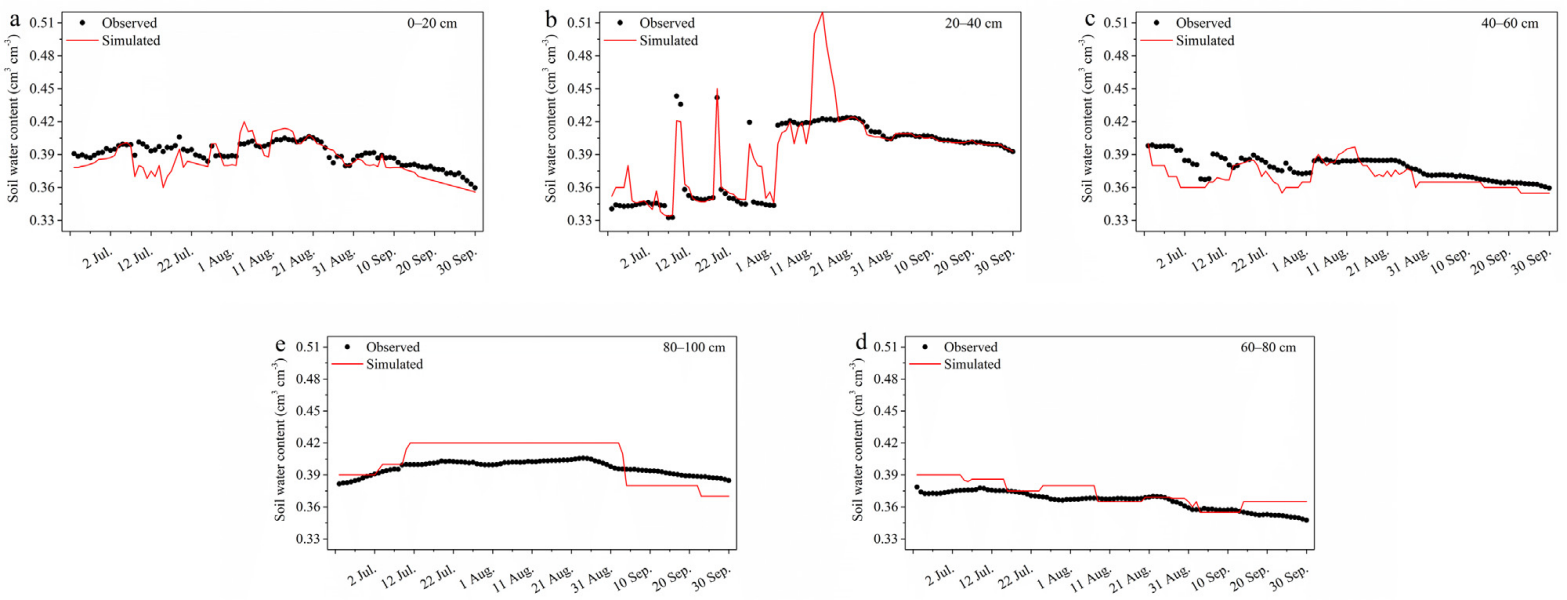
**The measured and simulated values of soil water at 0–20 cm (a), 20–40 cm (b), 40–60 cm (c), 60–80 cm (d) and 80–100 cm (e) layers**.

The differences between the measured and simulated unsaturated soil water contents were layer dependent (Fig. 6). Analyses of the dynamic characteristics of unsaturated soil water in each layer of the experimental area revealed that the unsaturated soil water content in the wetlands increased gradually from 0 to 40 cm as the depth of the soil layer increased, decreased at a depth of 40–80 cm, and increased at a depth of 80–100 cm. However, the change in unsaturated soil water decreased as the soil depth increased, and the response of the upper unsaturated soil water layer to precipitation was consistent.

## 4 Discussion

### 4.1 Spatiotemporal variations of isotopic compositions in waters

Changes in the abundance of water isotopes along with precipitation, large-scale circulation forms, and water vapour source paths are closely related to the initial state of the water vapour. Generally, smaller slopes and intercepts occur in areas with lower relative humidity and stronger unbalanced evaporation [29,30]. The slope and intercept of the LMWL were smaller than those of the GWML (Fig. 2a), which indicated the drought and semi-arid climate features of the MNNR. Because precipitation is subject to unbalanced secondary evaporation during the rainfall process in this semi-arid area, the isotope fractionation of the precipitation deviates from the GMWL due to evaporation, thus showing a decrease in slope and intercept.

Continuous or pulsed injection in soils of δD and δ^18^O in water by precipitation is controlled by the local climate, and the total infiltration amounts and rates are determined by the precipitation isotopic composition, intensity, and frequency and then affected by evaporation after infiltration in soils [31,32]. A marked peak of δ^18^O in groundwater that occurred on 7 September (Fig. 3b) was consistent with the enrichment in precipitation (Fig. 3a) and unsaturated soil water (Fig. 4b), which indicated that groundwater had been replenished by rainfall through unsaturated soil water downwards. However, the relatively marked depletion of isotopes in the groundwater (Fig. 2b) indicated that the isotopic composition of MNNR was not only recharged by local precipitation via unsaturated soil water infiltration, but also might have received other water sources off-site [33].

Although the inlet/outlet runoff and the overland flow were connected to each other and their isotopic compositions showed no clear boundaries between each other (Fig. 2a), the heavier δ^18^O in the outlet runoff confirmed the isotopic enrichment by evaporation when surface water flowed through the wetland (Fig. 2c-e).

### 4.2 Unsaturated soil water sourcing

Seasonal and vertical variations of δD and δ^18^O in unsaturated soil water can provide information about the water movement mechanism, because precipitation injects pulsed input of isotopic signals to soils downwards [7]. In the MNNR, the study site was seasonally flooded with surface water. We expected that the wetland soil would receive overland flow replenishment either directly from local precipitation or indirectly from surface water. Our results showed that nearly all the δ^18^O of unsaturated soil water at all depths was consistently heavier than the precipitation and lighter than the groundwater (Fig. 2b), which indicated that the unsaturated soil water was not only sourced from overland flow, but also from groundwater [6,14].

In addition, the isotope variabilities in unsaturated soil water not only reflect the seasonality of precipitation infiltration but are also affected by groundwater evaporation when precipitation is discontinuous [10,31], and then form vertical isotope composition gradients. The unsaturated soil water lines (Table S2) indicated that the upper soil layer of 0–15 cm was mainly sourced from precipitation because they were close to the LMWL, while the groundwater could move up to the 25 cm layer that shared a similar slope of the water line with the groundwater [8]. Consequently, capillary rising groundwater could be considered for water replenishment in dry periods, as such groundwater is used to reach the root layer of some wetland plants [25].

The d-excess reflects the isotopic composition of water vapour in the atmosphere and depends on the status of the water vapour evaporation source [30]. Previous studies near Northeast China confirmed that d-excess can be used as an effective tracer for estimating the mean residence time of unsaturated soil water [32,34]. Unfortunately, limited by the field work conditions, the monitoring duration was too short to model δD, δ^18^O, or d-excess [31,32,34], and the mean residence time could not be evaluated further. However, the temporally changed d-excess in this study indicated a half-sinusoidal period without significant vertical variation (Table S3, Fig. 4c). Longer isotopic data for different waters are required for further water cycling studies in the MNNR.

### 4.3 Vertical distribution and movement of unsaturated soil water

Soil properties determine the movement of event and pre-event water. In piston flow occurring in a homogeneous soil matrix, the pre-event unsaturated soil water is pushed by event water downward; however, in preferential flow occurring in heterogeneous soil, the event water can bypass the pre-event water [6,8]. The crossed profiles of δD and δ^18^O (Fig. 3a, b) and fluctuating trends of the mean δD and δ^18^O with soil depth (Fig. 5a) suggested that vertical reciprocating movement would likely occur seasonally because such characteristics of isotopic vertical distribution with depth did not match the unidirectional piston or preferential flow features [6].

The unsaturated soil water changes at different depths during different periods were visually represented by amplitudes and mean values, which could be reflected by the CV as a quantitative indicator [35]. Unsaturated soil water in shallow layers had larger CV values due to external factors, such as surface climate, hydrological conditions, and vegetation cover on the surface; however, unsaturated soil water in deeper layers was relatively less affected by external climate conditions (Fig. 5b).

Most soil layers showed weak variability during different periods. However, with increasing soil depth, the variability gradually decreased, as deep soils were not easily affected by external climatic conditions. From June to July, the lower changes in unsaturated soil water were due to the relative lack of surface water, while in August, farmland drainage began to enter the wetlands, and the wetland unsaturated soil water content increased and began to stabilise in September (Fig. 6).

### 4.4 Implication for wetland water-saving restoration

For semi-arid and sub-humid areas, the economic and efficient restoration of degraded wetlands is a key challenge for implementing relevant international conventions and improving local ecological well-being [13]. Coupled with field observations and model simulations, we expected to explore the optimal water replenishment timings, and then support the water-saving restoration plans and practices of degraded wetlands due to water shortage.

The seasonal distributions of precipitation and evaporation of the MNNR (Fig. 1b) suggest that 82% of the annual precipitation and 47% of the evaporation occurred during the growing season (June–September). Therefore, if the precipitation in the growing season is significantly lower than average and the input surface runoff is reduced due to natural or anthropogenic factors, such wetlands are prone to drought and emergency water supply is necessary. Replenishing water is the primary issue of local wetland restoration. Based on the model predicting the characteristics of unsaturated soil water changes throughout the entire growing season combined with the changes in surface water and groundwater in the study area (Fig. 6), wetland vegetation was likely to be in a water-deficient state from the end of June to the beginning of July, which was an important time for maintaining the reasonable water requirements of the wetland.

In addition, given the bidirectional movement of unsaturated soil water (Fig. 5), groundwater and soil water could be considered as alternative water pools for wetland plants, especially under suitable soil and hydrometeorological conditions. The practical use of such unconventional water resources requires further investigation.

## 5 Conclusion

Wetlands located in semi-arid areas are usually susceptible to water shortages and are subject to degradation risks. The effective replenishment of such wetlands in degraded dilemmas is particularly important for sustaining their ecological functions. In this study, we concluded that unsaturated soil water sourced from both precipitation and groundwater with variations in different layers and bidirectional unsaturated soil water movement highlighted the possibility of utilising capillary rising groundwater to sustain wetland plants’ water requirements when suffering water shortages. Local ecological water replenishment was conducted from the end of June to the beginning of July as the critical time for restoring degraded wetlands with limited water resources.

## Declaration of competing interest

The authors declare that they have no conflicts of interest in this work.

## Appendix Supplementary materials

Image, application 1
