# Supplementary material for: Isotopic evidence for soil water sources and reciprocal movement in a semi-arid degraded wetland: Implications for wetland restoration
Source: Fundam Res. 2022 Nov 17;3(6):861–7. doi: 10.1016/j.fmre.2022.11.001 (PMC11197564; doi:10.1016/j.fmre.2022.11.001)
Supplement: Supplementary file 1 [file mmc1.docx]

**Supplemental materials**

**Table S1: One-way analysis of variance (ANOVA) of temporal change on the δD and δ^18^O from the precipitation, groundwater, overland flow, and inlet and outlet runoff.**

|  |  | Precipitation | Groundwater | Overland flow | Inlet runoff | Outlet runoff |  |
| --- | --- | --- | --- | --- | --- | --- | --- |
| δD | *F* | 16.972 | 0.994 | 4.447 | 1.771 | 7.941 |  |
|  | *p* | <0.001 | 0.432 | 0.004 | 0.165 | <0.001 |  |
| δ^18^O | *F* | 12.608 | 6.448 | 10.282 | 2.966 | 15.436 |  |
|  | *p* | <0.001 | 0.001 | <0.001 | 0.037 | <0.001 |  |

**Table S2: Local unsaturated soil water lines (LUSWL) linearly fitted by the δD and δ^18^O at each soil layer.**

| Soil depth | LUSWL |
| --- | --- |
| 5 cm | δD=3.99δ^18^O-31.48 (R^2^=0.73, p<0.01) |
| 15 cm | δD=5.13δ^18^O-20.07 (R^2^=0.90, p<0.01) |
| 25 cm | δD=1.92δ^18^O-48.24 (R^2^=0.27, p<0.05) |
| 35 cm | δD=4.25δ^18^O-32.90 (R^2^=0.84, p<0.01) |
| 45 cm | δD=2.17δ^18^O-47.53 (R^2^=0.47, p<0.01) |
| 55 cm | δD=3.45δ^18^O-35.64 (R^2^=0.60, p<0.01) |
| 65 cm | Non-significant |
| 75 cm | δD=4.10δ^18^O-32.92 (R^2^=0.95, p<0.01) |
| 85 cm | δD=3.20δ^18^O-37.97 (R^2^=0.95, p<0.01) |
| 100 cm | δD=2.41δ^18^O-46.05 (R^2^=0.74, p<0.01) |

**Table S3: Two-way ANOVA of temporal change and soil layer on the δD, δ^18^O and deuterium excess (d-excess) from the unsaturated soil waters.**

|  |  | Time | Soil layer | Time×Soil layer |
| --- | --- | --- | --- | --- |
| δD | *F* | 0.799 | 3.723 | 1.877 |
|  | *p* | 0.554 | 0.001 | 0.009 |
| δ^18^O | *F* | 15.341 | 3.046 | 1.812 |
|  | *p* | <0.001 | 0.003 | 0.013 |
| d-excess | *F* | 40.854 | 1.575 | 1.360 |
|  | *p* | <0.001 | 0.136 | 0.125 |

**Table S4: Mean values of soil bulk density and texture compositions for each layer used for HYDRUS model.**

| Soil layer  (cm) | Bulk density  (g cm^-3^) | Clay  (%) | Loam  (%) | Sandy  (%) |
| --- | --- | --- | --- | --- |
| 0**–**20 | 1.49 | 2.53 | 18.68 | 78.79 |
| 20**–**40 | 1.47 | 5.71 | 25.93 | 68.36 |
| 40**–**60 | 1.50 | 8.25 | 36.25 | 55.49 |
| 60**–**80 | 1.49 | 8.45 | 36.66 | 54.89 |
| 80**–**100 | 1.48 | 8.81 | 39.16 | 52.04 |

**Table S5: The unsaturated soil water characteristic curve parameters after model identification. *Qr* and *Qs* represent the remaining water content (cm^3^ cm^-3^) and saturated water content (cm^3^ cm^-3^), respectively; *α* is an empirical parameter related to the air inlet pressure value; *n* is the pore size distribution (dimensionless); *l* is an empirical shape parameter (dimensionless); *Ks* is the saturated hydraulic conductivity.**

| *Qr* (cm^3^ cm^-3^) | *Qs* (cm^3^ cm^-3^) | *α* (l m^-1^) | *n* | *Ks* (cm d^-1^) | *l* |
| --- | --- | --- | --- | --- | --- |
| 0.0368 | 0.3784 | 0.0451 | 1.7651 | 128.65 | 0.5 |
| 0.0358 | 0.3777 | 0.0369 | 1.4803 | 65.48 | 0.5 |
| 0.0366 | 0.3601 | 0.0220 | 1.4307 | 33.61 | 0.5 |
| 0.0371 | 0.3619 | 0.0209 | 1.4359 | 33.68 | 0.5 |
| 0.0376 | 0.3590 | 0.0180 | 1.446 | 29.79 | 0.5 |

**Table S6: Comparisons between the measured and stimulated unsaturated soil water contents. RMSE, root mean square error; R^2^, determination coefficient.**

| Soil layer (cm) | RMSE (cm^3^ cm^-3^) | R^2^ |
| --- | --- | --- |
| 0**–**20 | 0.00897 | 0.81 |
| 20**–**40 | 0.01964 | 0.86 |
| 40**–**60 | 0.00822 | 0.68 |
| 60**–**80 | 0.00714 | 0.77 |
| 80**–**100 | 0.00996 | 0.85 |
